# Cellular nanointerface of vertical nanostructure arrays and its applications

**DOI:** 10.1039/d1na00775k

**Published:** 2022-02-21

**Authors:** Aihua Zhang, Jiaru Fang, Xiangling Li, Ji Wang, Meiwan Chen, Hui-jiuan Chen, Gen He, Xi Xie

**Affiliations:** State Key Laboratory of Optoelectronic Materials and Technologies, Key Laboratory of Display Material and Technology, School of Electronics and Information Technology, Sun Yat-Sen University Guangzhou 510006 Guangdong Province China xiexi27@mail.sysu.edu.cn; The First Affiliated Hospital of Sun Yat-Sen University Guangzhou 510080 China; Institute of Chinese Medical Sciences, University of Macau Taipa Macau SAR China; Key Laboratory of Molecular Target & Clinical Pharmacology, State Key Laboratory of Respiratory Disease, School of Pharmaceutical Sciences & The Fifth Affiliated Hospital, Guangzhou Medical University Guangzhou 511436 P. R. China; School of Biomedical Engineering, Sun Yat-Sen University Guangzhou 510006 China

## Abstract

Vertically standing nanostructures with various morphologies have been developed with the emergence of the micro-/nanofabrication technology. When cells are cultured on them, various bio–nano interfaces between cells and vertical nanostructures would impact the cellular activities, depending on the shape, density, and height of nanostructures. Many cellular pathway activation processes involving a series of intracellular molecules (proteins, RNA, DNA, enzymes, *etc.*) would be triggered by the cell morphological changes induced by nanostructures, affecting the cell proliferation, apoptosis, differentiation, immune activation, cell adhesion, cell migration, and other behaviors. In addition, the highly localized cellular nanointerface enhances coupled stimulation on cells. Therefore, understanding the mechanism of the cellular nanointerface can not only provide innovative tools for regulating specific cell functions but also offers new aspects to understand the fundamental cellular activities that could facilitate the precise monitoring and treatment of diseases in the future. This review mainly describes the fabrication technology of vertical nanostructures, analyzing the formation of cellular nanointerfaces and the effects of cellular nanointerfaces on cells' fates and functions. At last, the applications of cellular nanointerfaces based on various nanostructures are summarized.

## Abstract 2

The fabrication technology of vertical nanostructures, the formation of cellular nanointerfaces and the effects of cellular nanointerfaces on cells' fates and functions, as well as its applications.

## Graphical abstract

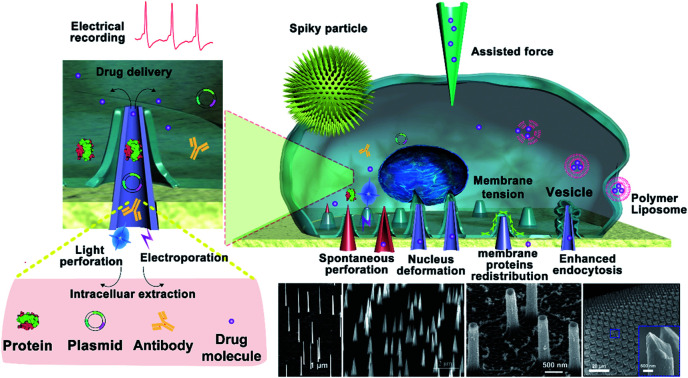

## 1. Introduction

The cell membrane is a tightly regulated system delineated by a complex and active lipid bilayer.^1–3^ When cells are cultured on different nanostructures, the close contact between the cells and nanostructures results in unique cellular interfaces correspondingly. When nanostructures are fabricated with the final aim of coupling them with biological cells, the most critical feature of the cell–nanostructure interface is the connected reaction between the cytomembrane and the nanostructure surface.^4,5^ When cells settle on a vertical nanostructure, the part of the cell that touches the vertical nanostructure bends and wraps around the nanostructure under its own gravity.^6,7^ Thus, a cellular nanointerface is formed between the cell and the vertical nanostructure. The morphology of the cell would change with the shape of the nanostructure, and the various cell morphologies trigger a series of reactions between intracellular materials (such as proteins, RNA, DNA, enzymes, *etc.*).^8–12^ The dimensions of nanostructures could be prepared from tens of nanometers to a few micrometers, where nanostructures with vertical features may play a critical role in cellular manipulation and bio-cargo delivery. When nanostructures come in contact with micron-sized cells, a highly localized interaction will be created at the cellular nanointerface. Most of the vertical nanostructures possess 1D features, which are between 10 nm and 2 μm in diameter and usually several micrometers in height. When the diameter of the vertical nanostructures is less than 100 nm, it is possible for the cells to be spontaneously perforated under its own cellular forces.^9,13^ The existence of spontaneous cell perforation remains controversial, as the cell's own gravity does not seem to be sufficient to cause it.^14^ When the vertical nanostructures are a few hundred nanometers in size, the cell membrane is not easily perforated spontaneously and would rely on external forces for cell penetration. According to a computational model of cell perforation and a large number of experiments, when the cell is completely deformed around the vertical nanostructure, there is certain cellular tension in the part of the cell in contact with the nanostructure. In addition, the nanostructures can make the external forces more localized on cells, or when physical stimulations such as the mechanical force, heat or electric fields are coupled, the highly local interactions enable these stimulations to be more focused and more directed on cells. For example, bulk electroporation often requires hundreds of volts or even thousands of volts to cause cell perforation, yet electroporation based on nanostructures usually requires only tens of volts.^15^ Therefore, the cellular nanointerface can enhance the effects of physical stimulations coupled to the vertical nanostructures, reducing the required conditions to act on cells compared to other conventional methods. The nano-scale trauma on the membrane created by the nanostructures or the coupled stimulations could be well controlled to be little perturbations to the cell, thus the cell membrane can complete self-repairing after stimulation. At the same time, a cellular nanointerface has been found to perturb cells minimally even in postdelivery culture.^16–18^ These advantages enable vertical nanostructures to interface with a wide variety of cells for cellular regulation, such as on primary immune cells and cardiomyocytes.^19^

The effects of the cellular interface not only change the cells' morphologies but also change the intracellular materials and genes. In addition, cellular behavior and functions including the proliferation, apoptosis, differentiation, immune activation, *etc.* will also be affected. Vertical nanostructures generally include the features of randomly distributed 1D features or a well-aligned nanoneedle array. When the cells come in contact with nano-needle arrays initially, the nano-needle arrays with different densities and modifications can also cause different cell behavior changes, such as adhesion,^20,21^ diffusion, and migration,^22,23^ which need systematic study. Therefore, cells can be artificially regulated or promoted into specific functional cells by designing nanostructures with different structures and functions,^24^ which could be further applied for applications such as regulating cell signal transmission, guiding cell migration,^25,26^ stimulating stem cell differentiation,^27,28^ promoting the maturation of osteoblasts, regulating the activity of macrophages, *etc.* Measurements of biomolecules inside cells usually require the insertion of nanoprobes (mainly as solvated, in the solid state, or as photonic crystals) through the cell membrane, or placing the nanoprobes very close to the cell membrane. In addition, the rapid developments in biological therapies and gene editing technology requires the delivery of bio-cargos across the cytomembrane to different subcellular locations, as well as the nucleus and mitochondria. The technique of nano-perforation based on vertical nanostructures can not only manipulate complex biological phenomena in a rigorous and reproducible manner but also can transport cargos into the targeted intracellular space effectively.^29^ More importantly, the nano-perforation technique might produce less cellular perturbations than conventional viral, biochemical, and bulk electroporation techniques, which might serve as effective tools in intracellular cargo delivery, gene editing, and biological therapy for biomedical applications.^30,31^ The nano-perforation technique not only realizes the accurate and efficient transport of bio-cargos into cells but also provides a strong foundation for oriented gene transfection and directional cell modification.

**Fig. 1:**
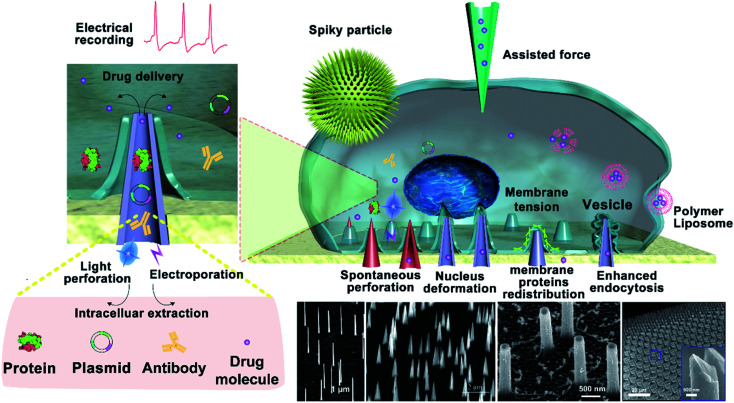
Illustration of the cellular nanointerface of vertical nanostructure arrays and its applications. Various bio–nano interfaces between cells and vertical nanostructures would cause a series of cellular reactions, including the increase of cytomembrane tension, the redistribution of membrane proteins, and the release of vesicles. Spontaneous or force-assisted cell perforation at the cellular interface with different vertical nanostructures possesses multiple promising applications, including intracellular substance detection, drug delivery, and recording of intracellular and extracellular electrical signals.

In general, the spontaneous perforation of the cytomembrane on the cellular nanointerface occurs rarely.^32,33^ Usually, it requires external forces or chemical modification^34^ to cause cellular perforation, while the external forces are often divided into electroporation,^35^ photoporation,^36,37^ and mechanical perforation (ultrasound power, external pressure, *etc.*).^12,38^ Only when the vertical nanostructure is sufficiently sharp, the cytomembrane could be perforated spontaneously.^39^ Spontaneous or force-assisted cell perforation at the cellular interface with different nanostructures possesses versatile applications. For example, it could enable intracellular substance detection,^40,41^ drug delivery,^42,43^ recording of intracellular and extracellular electrical signals, and real-time monitoring of intracellular biochemical signals (mainly proteins, metabolites, lipids, enzymes, and other substances in the intracellular environment).^44–46^ Among them, taking mechanical force conduction on the cytomembrane as an example, most nanomaterials contact exclusively with the cytomembrane and generate a mechanical force at the cellular interface that causes the cytomembrane to bend. Some intracellular proteins recognize the changes in the membrane curvature and then regulate cellular signals and activate cell pathways,^47,48^ and some organelles' structures and functions will be changed through established mechanosensory signal transduction,^49^*etc.* (Fig. 1).

In summary, the study of cell interface behavior is of great significance for decoding the interactions between biological cells and artificial nanostructures. Here, we first summarize the preparation technologies of vertical nanostructures, especially with nanoneedle-like features. The effects of different cellular nanointerfaces on the cell morphology, behavior, function, and fate during cell culture at different vertical nanostructures were introduced emphatically. Then, we summarize in detail the cellular behavior and application before and after cell perforation at the cellular interface, including intracellular bimodular detection, extracellular cargo transportation, gene transfection, and cellular immune modification, as well as the recording of intracellular and extracellular electrical and biochemical signals. Studies on the interaction between cells and different vertical nanostructure interfaces not only provide a great guidance value for the application of cellular nanointerfaces in cell biosensors and the biomedical field^50,51^ but also establish the basis for understanding the physical interactions of cells with artificial structures on the cellular level, which would facilitate the development of new technologies for cell (stem cells, immune cells, *etc.*) modification^52^ and cell recording on functional nerve cells in the field of neurology.^53,54^

## 2. Fabrication of vertical nanostructures

**Fig. 2:**
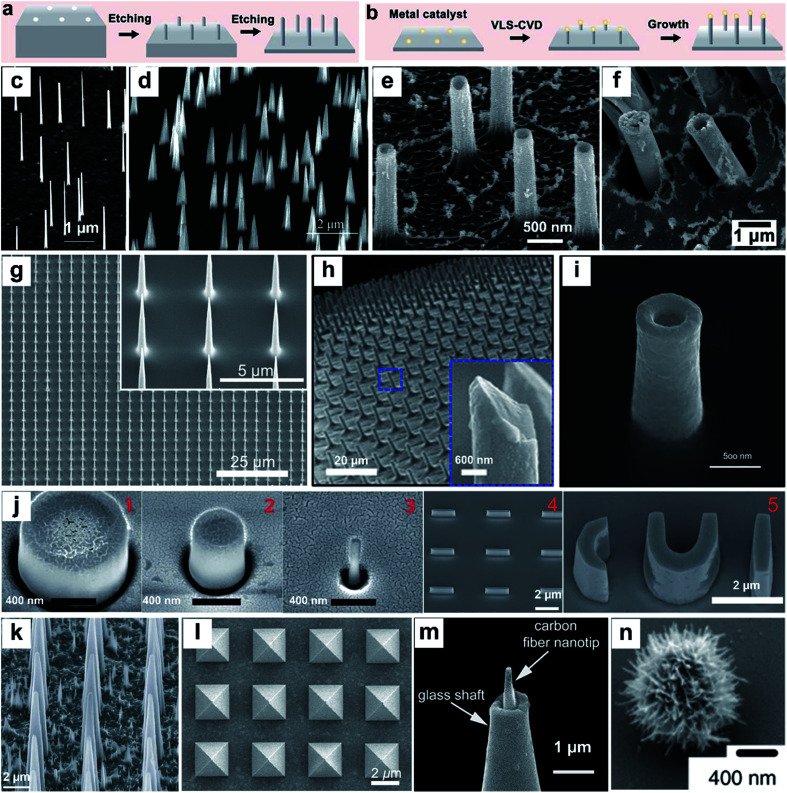
Schematic of top-down (a) and bottom-up (b) approaches for vertical nanostructure array fabrication and their cellular applications. (c–n) SEM images of several different vertical nanostructure arrays: (c) nanowires,^65^ (d) nanotips,^73^ (e) Al_2_O_3_ nanostraws,^67^ (f) Pt nanostraws,^16^ (g) nanoneedles,^68^ (h) Si nanoneedles,^69^ (i) nanoelectrode,^70^ (j) nanopillar, nanobar, and nano-CUI structure,^71^ (k) porous silicon nanoneedles,^72^ (l) pyramids,^59^ (m) carbon fiber nanometric electrode,^73^ and (n) spiky particles.^74^ Reprinted with permission Copyright: 2007, Nano Letters; 2014, Angewandte Chemie International Edition; 2013, ACS nano; 2019, ACS Appl. Mater. Interfaces; 2019, Advanced Materials; 2018, Science Advances; 2017, Scientific Reports; 2017, Nature Nanotechnology; 2015, Nature Materials; 2017, ACS Nano; 2014, Angewandte Chemie International Edition; 2018, Nature Nanotechnology.

Nanostructures can be prepared from a wide range of materials sources, including metals, inorganic nonmetals, polymer materials, and various hybrid materials.^55–57^ There are multiple routes for the fabrication of vertical nanostructures. In general, they can be divided into the bottom-up approach or the top-down approach,^58^ or a combination of them.^59^ The bottom-up approach is a form of synthesis that ranges from small to large, with atoms or molecules stacked in a certain direction to produce nanostructures with a high-aspect ratio (Fig. 2a). The vapor–liquid–solid (VLS) mechanism, the fluid–liquid–solid (SFLS) mechanism,^60^ the supercritical fluid–solid–solid (SFSS) mechanism,^61^ the solution–liquid–solid (SLS) mechanism,^62^ the vapor–solid–solid (VSS) mechanism,^63^ and the oxide assisted growth (OAG)^64^ mechanism are the commonly used bottom-up routes for the production of vertical nanostructures. Using the metal–organic vapor-phase epitaxy (MOVPE) technique for nanostructure growth enabled the production of epitaxial nanostructures^65^ (Fig. 2c). The development of the chemical vapor deposition (CVD) technique and the atomic layer deposition (ALD) technique allow the epitaxial growth of nanostructures with a specific template^66,67^ (Fig. 2d and e). The electrodeposition technique does not rely on the use of valuable instruments, and it is convenient to prepare metal nanostructures^16^ (Fig. 2f). The top-down routes often need various etching techniques (Fig. 2b), usually including lithography etching,^68^ reactive ion etching (RIE),^69^ and focused ion beam etching (FIB)^70^ (Fig. 2g–i). Generally, electron-beam lithography often uses various masks with different designed shapes to obtain nanostructures with corresponding shapes^71^ (Fig. 2j).

With the development and maturity of the micro-/nanofabrication technology, the fabrication of vertical nanostructures is not limited to the simple bottom-up or top-down model. A combination of bottom-up growth and top-down etching has been used widely as well. For example, Tasciotti's^72^ group synthesised a porous Si nanoneedle array by combining conventional microfabrication and metal-assisted chemical etching (MACE) together (Fig. 2k). The technique of photolithography templates proposed by Mazur's group^59^ also combined the e-beam lithography and the deposition technique together, while the template's shape can be designed manually and the nanostructures with the corresponding morphology can be fabricated subsequently (Fig. 2l). In addition to the micro-/nanofabrication approaches, nanostructures can also be fabricated by other ways, such as pulling a glass capillary to a sub-micropipette and flame etching to a needle shaped nanotip (Fig. 2m),^73^ and by hydrothermal growth of spiky particles (Fig. 2n).^74^ The wide range of fabrication methods leads to the diversity in physical properties of vertical nanostructures, affording them applications in a vast network of research fields, such as fundamental cellular studies,^56,75–77^ nanoelectrode-based electrophysiology,^78,79^ biosensing,^80^ mechanotransduction,^81^ and intracellular delivery.^82–85^ Nanostructure-based platforms generate unique nano-topographical cues for cell perturbation. Here, we summarize and emphasize the performance of vertical nanostructure arrays of different geometries (nanoneedle, nanopillar, nanowire, nanotip, nanostraw, spiky particle, *etc.*, see Fig. 2c–n) in orchestrating cell functions, behaviors, intracellular drug delivery or extraction, and fate conversions through experimental and theoretical studies.

## 3. Behavior of the cellular interface

### 3.1 Structural behavior of cells

When cells are cultured on nanostructure arrays with a convex surface, the interaction between the cells and the topological surface of the nanostructures leads to the shape changes of cells, nuclei, or organelles. The main manifestation is the cell bending and deforming under the induction of vertical nanostructures, where the cells' deformation is mainly attributed to the redistribution of membrane proteins, especially the proteins associated with cells’ curvature such as kinesin and clathrin. The redistribution of cells' curvature induces cells' bending and encapsulating around the protruded nanostructures. At the same time, the nanometer structures induce the curvature associated actin polymerization on the cell membrane. The aggregation of curvature-sensitive proteins increases the clathrin-mediated endocytosis. Finally, the cell membrane will tightly wrap around the nanostructures, forming a conformation of the cell membrane and even causing the nucleus to bend and deform, while the integrity of the membrane could still be preserved.^5^ In this section, we summarized in detail the influence of cell bending and deformation induced by vertical nanostructures that interfaced with cells without cell penetration.

#### 3.1.1 Cytomembrane bending

**Fig. 3:**
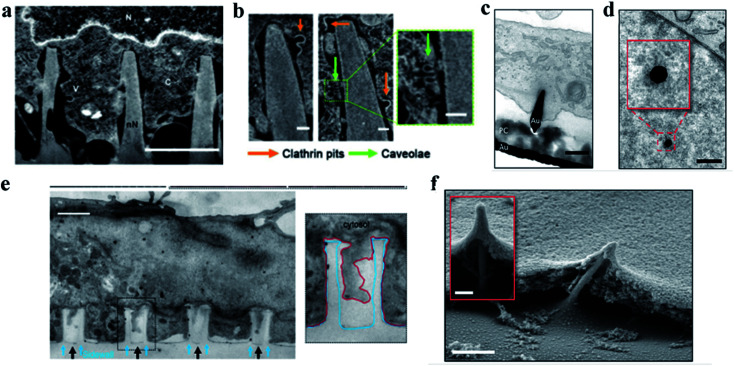
(a) Representative FIB-SEM image of an orthogonal cross section of an hMSC on nanoneedles after 6 h of interfacing; scale bar, 2 μm.^86^ (b) FIB-SEM image of the interface showing two classes of endocytic vesicles accumulating around nanoneedles: clathrin pits (orange arrows) and caveolae (green arrows); scale bar, 100 nm.^86^ (c) TEM cross section image of a cell protrusion extending to the electrode's PC cone and thereby tightly enclosing the penetration site. The basal membrane sticks closely to the PC layer (PC) and ruptures close to where the PC substrate and the gold electrode (Au) join; scale bar, 200 nm.^87^ (d) TEM horizontal section image of a nanoelectrode penetrating through the nuclear envelope in an area without any ultrastructural reorganization of heterochromatin. The inset shows a higher magnification of the chromatin–electrode interface; scale bar, 300 nm.^87^ (e) TEM vertical section image of a cardiomyocyte growing on top of quartz nanotube arrays (black arrows), showing that the bottom plasma membrane protrudes into the nanotubes. The inset shows the outline of a nanotube (blue line) and the plasma membrane (red line); scale bar, 1 μm.^88^ (f) SEM image of a fractured cross section showing basal membrane penetration and the membrane electrode interface. The inset shows basal membrane-associated, filamentous structures anchoring the cell to the electrodes; scale bar in the larger image, 1 μm, and the inset, 200 nm.^87^ Reprinted with permission Copyright 2019, Advanced Materials; 2019, Nano Letters; 2014, Nature Communications.

Multiple characterization techniques such as scanning electron microscopy (SEM), transmission electron microscopy (TEM), focused ion beam/scanning electron microscopy (FIB-SEM), fluorescence and confocal fluorescence microscopy, *etc.* have proved that nanostructures with a protruding surface could induce cell membrane bending and deformation. As shown in Fig. 3a–f,^86–88^ by SEM characterization, the cytomembrane around the nanoneedles shows a complete lipid bilayer, accompanied by clathrin coated pits and caveolae. This proves that the cytomembrane was bent and tightly wrapped around the nanoneedle structures, as shown in Fig. 3a and b.^86^ When the cell comes in contact with the nanostructures, the cytomembrane will deform due to the cell's gravity.^39^ Cui's group studied the effects of the membrane curvature on cell behavior in detail. The shape and the position of the cellular nanointerfaces determine the precise curvatures of plasma membrane induced by the nanostructures.^89^ The membrane bending behavior produced local mechanical forces between the nanostructure and the cytomembrane, and many important cellular functions can be regulated by adjusting the mechanical forces at the cellular interface. For example, the mechanical force will assist the delivery of toxins *via* immunological synapses and enhance the killing of T cells.^90^ The local actin polymerization can drive membrane protrusion and control cell migration.^91^

#### 3.1.2 Nuclear bending

In addition to deforming the plasma membrane, some nanostructures can also cause deformation of the nuclear envelope.^37,92^ The corresponding characterization methods include SEM, FIB-TEM,^20,93^ Raman, and fluorescence spectroscopy.^94^ The karyotheca has a double membrane structure, with one side connected to the cytoskeletal network for mechanical transduction and the other side connected to the chromosome for gene expression and regulation. Therefore, the karyotheca deformation induced by vertical nanostructures can activate nuclear mechanical conduction and change the gene expression and cell behavior.^20^ The vertical nanostructure did not come in direct contact with the karyotheca before cell penetration; therefore, nuclear deformation caused by nanostructures must be mediated by intracellular forces.

**Fig. 4:**
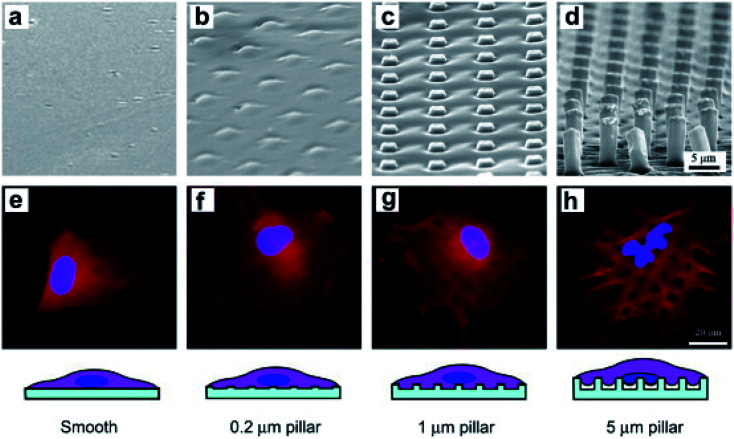
SEM micrographs of smooth and rough PLGA films of the marked heights of micropillars (a–d) and fluorescence micrographs of BMSCs on the corresponding films after 6 h of culture (e–h). The cells were stained to label the nuclei (blue) and filamentous actins (red).^99^ Reprinted with permission Copyright 2012, Biomaterials.

The cytoskeleton, especially F-actin, regulates the transfer of the mechanical force between nanostructures and the nucleus.^24,49,95^ Studies on nuclear deformation and mechanical coupling between the cell membrane and the nucleus showed that vertical nanostructure-induced nuclear deformation is determined by nuclear stiffness, as well as the opposing effects from actin and intermediate filaments. The nuclear membrane deformation induced by vertically protruding nanostructures can activate nuclear mechanical conduction, change the nuclear morphology, and even cause gene expression changes.^4,49,95–97^ For example, the Ca^2+^ ion channel activation and the cytoskeleton reorganization can cause the stem cells to differentiate preferentially.^98^ Different from the nuclear deformation caused by nanostructures, the nuclear deformation is characterized by the nucleolus depression of the nucleus.^20^ Furthermore, the depth, width, and curvature of nuclear deformation can be controlled by varying the geometry of nanostructures such as the diameter, density and length, as shown in Fig. 4a–h.^99^ Nuclear deformations generally do not significantly affect DNA replication and cell proliferation when the cells are cultured on a low aspect ratio nanostructure interface. This process was regulated by combining the uniform pressure applied by the actin cap and the localized pressure applied by the actin underneath the nucleus. The cell morphology and the nucleus shape interacted with each other to adapt to the microenvironment of vertical nanostructures.^96^

The actin cytoskeleton powers the bending of the karyotheca. The process of karyotheca bending can be divided into three stages: stage 1, mechanical bending; stage 2, partial recovery of actin, and finally stage 3, the geometry of the nucleus responding to the microcolumn array in an overshoot manner. Ding's group fabricated polymeric micropillar arrays with appropriate dimensions to induce severe self-deformation of cell nuclei and investigated how the nuclear shape changed over time.^49,95^ The results showed that the nuclei of mesenchymal stem cells (MSCs) on the poly(lactide-*co*-glycolide) (PLGA) micropillars exhibited significant initial deformation, followed by partial recovery. The treatment of cytochalasin D suppressed the recovery of nuclei, which indicated the involvement of the actin cytoskeleton in regulating the recovery at the second stage of nuclear deformation.^100^ Some low aspect ratio nanostructures can even promote cell proliferation or division. However, high and pointed nanostructures with a high aspect ratio (length/diameter > 10) can cause large deformation of cells, reducing the cell proliferation rate and leading to the appearance of multinucleated cells.^4,97^ The generation of ROS (reactive oxygen species) and DNA damage can even be observed.^8,10^ Therefore, the high curvature of nuclear membrane deformation may affect signal transmission and gene expression in the nucleus.^89^

#### 3.1.3 Cell adhesion

**Fig. 5:**
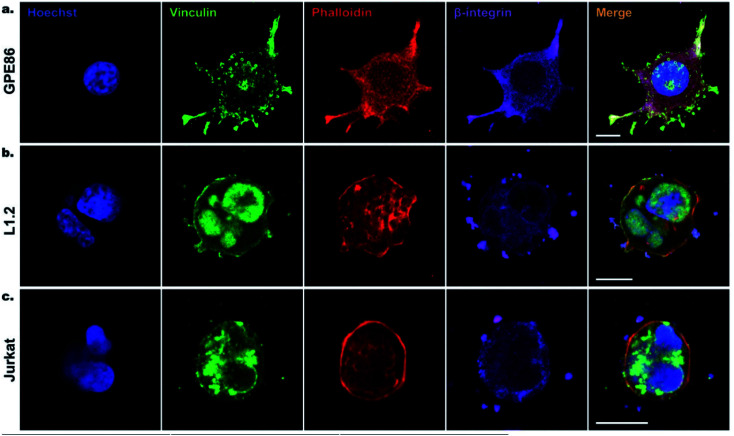
Confocal imaging of cell focal adhesion formation on SiNWs. (a) GPE86, (b) L1.2, (c) Jurkat cells after 6 h of incubation on SiNWs. Cell nuclei stained with Hoechst (blue), vinculin (green), phalloidin (red), and β-integrin (purple) were used to reveal the cytoskeleton and focal adhesion points on SiNWs. Scale bars, 10 μm. Reprinted with permission Copyright 2019, Small.

The cells settled on the vertical nanostructures under the gravity after their contact with the nanostructures, and the interaction of the actin on the cell membrane with the nanostructures make the cells adhere to the nanostructures,^101^ as shown in Fig. 5a–c.^12^ The adhesive condition of cells on nanostructures mainly depends on the interaction intensity, and it changes over time. The intensity of cellular interaction between the cell and the nanostructures not only depends on the nanostructures' properties such as the density, size, shape, stiffness and the modification but also relies on the cell types and sizes.^56,102^

Chen's group studied the equilibrium state of cells' adhesion by analyzing the changes in free energy during adhesion. Theoretical results showed that the cell adhesion mode is actually determined by the balance between the adhesion energy and the deformation energy of the cell membrane.^21^ The protruding part of the nanometer needle provides abundant adhesion points for the filamentous pseudopodia of the cell, which can not only limit the cell diffusion but also enhance the adhesion force and reduce the cell shedding.^30^ In the sparse nanoarrays, the cell adhesion would increase, and the cytomembrane further bends and even penetrates into the hole of the nanoneedles.^88^ These cells seal the nanoneedles tightly around, which facilitate the nanoneedles to penetrate the cells steadily over time. When the cytomembrane is ruptured by external forces, focal adhesion complexes (FACs), filamentous actin and actin cortical pores will be formed on the cytomembrane.^87^

The dense nanoarrays inhibit the cells' adhesion between the cytomembrane and the cellular matrix rapidly, which cause the cells to sit on top of the dense nanoarrays.^30^ For example, Slomianny's group prepared vertically aligned silicon nanowire (Si NW) arrays that are modified with octadecyl trichlorosilane (OTS) to fabricate a micropatterned superhydrophilic/superhydrophobic Si NW surface. Then, Chinese Hamster Ovary K1 (CHO) cells were cultured on these patterned superhydrophilic/superhydrophobic Si NW surfaces. They found that the CHO cells adhered to the superhydrophilic regions selectively, while CHO cell adhesion was suppressed on the superhydrophobic surface.^103^ In addition to cell adhesion, cytomembrane bending can also cause a variety of protein rearrangements and deformations, such as the sensitive protein rearrangement, plasma membrane's inward deformation, *etc.* The inward deformation of the membrane resulting from actin contraction can result in the reassembly and even the puncture of the curved plasma membrane.^9,32^ Cells of different types have different morphologies and adhesion properties on the nanostructure arrays.^12^ Moreover, the endocytosis activity is closely associated with the fluidity of the cytomembrane. The degree of endocytosis is related to the size of the vertical nanostructures interfaced with cells and their geometric morphology (density, diameter, *etc.*). For example, with high density nanoneedles, the cells are completely jacked up and suspended. At low densities, the cell membrane comes into contact with the substrate, allowing the nanoneedle to be completely engulfed through endocytosis.^24,104^

#### 3.1.4 Cell migration

**Fig. 6:**
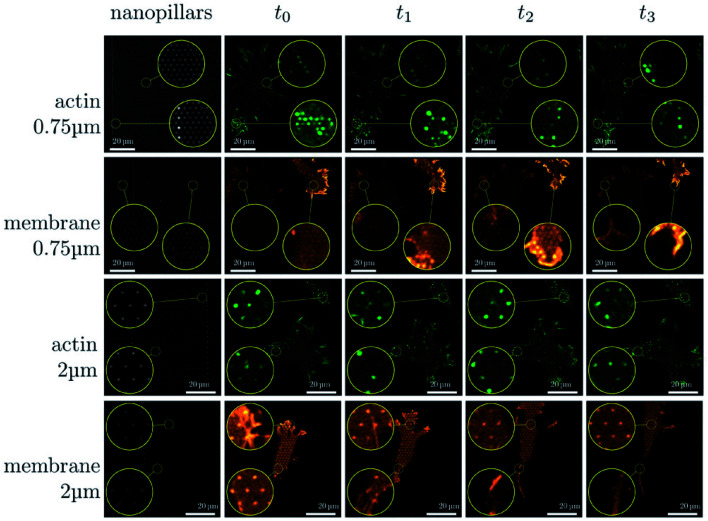
Sequence of images illustrating the dynamical changes in F-actin and the membrane configuration in fibroblasts migrating on dense and sparse nanopillar arrays. Circular insets show a magnified view of the same sample region for the image sequence in each row. Images presented are representative of *n* ≥ 50 cells for both dense and sparse arrays.^24^ Reprinted with permission Copyright 2019, Small.

The cells' adhesion on the nanostructures is the first step for the cellular response to the nanointerface and other fundamental functions.^12^ The protruding parts of nanostructures provide abundant adhesion points for cells' filopodia, which enhance the adhesion forces, diminish the cells' detachment, and restrict the cell spreading.^102^ Therefore, the cells' spreading would slow down when they adhered to the nanostructures. Cells' migration involves numerous steps, such as the protrusion of pseudopodia, the formation of new adhesions, the development of traction forces, and the release of old adhesions. In a mobile cell, the lamellipodia and filopodia extend from the main cell body as protrusions and form new focal adhesions in the direction of cell movement.^105^ Once the initial adhesion and the subsequent spreading occurred, the cell migration would start. The cell migration rate depends on many factors, such as the size and density of nanoarrays, the length and modification of the protruding nanostructures, the type and number of cells, *etc.* Sikorski has proven that, on the dense arrays, cells would stay at the top of the array, and the cells' migration was not significantly hindered, while cells' migration was significantly reduced on the sparse arrays. The migration of cells on sparse nanoarrays was also suppressed compared to that on the plane base, as shown in Fig. 6.^24^ Cells’ migration is related to the actin on the cytomembrane, and the actin recruitment was transient on both dense and sparse nanoarrays. While on the sparse nanoarrays, F-actin is assembled for a longer time,^12,101^ so that the migration of cells on sparse nanoarrays was restricted on the sparse nanoarrays.

The fabrication of nanostructure arrays with different geometric shapes or chemical modifications can be used to induce directional cell migration.^28,106,107^ P. Spatz's group prepared a vertical gold nanoelectrode interface with low impedance. This bioelectrical interface was able to penetrate the cytomembrane with the assistance of cellular adhesion. Cell adhesion reduced dramatically on the nanoelectrode passivated by PLL-PEG. At the same time, semi-adherent cells with morbid morphologies occurred, while there was no cell adhesion and spreading on the uncoated PC surface.^108^ Han's group constructed polydimethylsiloxane (PDMS) micropillar arrays with anisotropic adhesion properties. The arrays not only provide a strong guidance for cell migration on the nanoarray surface but also promote anisotropic cell adhesion.^109^ Researchers suggest that there are two mechanisms of cell migration *via* gene regulation: one is by suppressing the cell's spreading and the other is by promoting the cell's adhesion. Although the mechanism of gene regulatory progress is comprehended, detailed studies are urgently required to elucidate the interacting mechanism of nanoarrays with not only cells but also dynamic cellular components (*e.g.*, microvilli, filopodia, and lamellipodia).^56^

#### 3.1.5 Cell differentiation

**Fig. 7:**
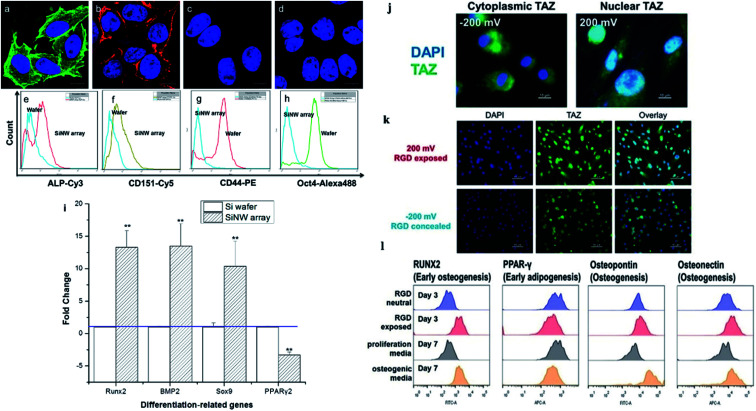
(a) Osteogenic and (b) chondrogenic differentiation of MSCs on Si NW arrays. MSCs cultured on a flat Si wafer for (c) osteogenic and (d) chondrogenic differentiation. (e–h) Flow cytometry analysis of ALP, CD151, CD44, and Oct4 after MSCs were incubated on the wafer and the Si NW array for 10 days. (i) Expression of genes related to osteogenesis, chondrogenesis, and adipogenesis as evaluated by qPCR.^48^ (j–l) Stem cell differentiation patterns induced with applied voltages. (j) Voltage-mediated morphology change further affects the expression of Hippo transducer TAZ. (k) Representative fluorescent images of hMSCs on the RGD/sulfonate surface with different voltages. (l) Flow cytometric analysis of osteogenic differentiation triggered by the RGD exposed surface.^22^ Reprinted with permission Copyright 2013, ACS Applied Materials & Interfaces; 2019, Angewandte Chemie.

In the process of stem cells' differentiation, the curvature-sensitive proteins on the cell membranes are localized, induced by the protruding nanostructures to promote the growth of protuberances and the differentiation of stem cells. A present study in Yang's group showed that the adhesion, proliferation, and differentiation of MSCs cultured on vertically aligned silicon nanowire (Si NW) arrays were significantly different from those on a flat silicon wafer and on control substrates. The interactions at the nanointerfaces between MSCs and the Si NW arrays caused the stem cells to preferentially differentiate toward osteocytes and chondrocytes, but not adipocytes, in the absence of supplementary growth factors, as shown in Fig. 7a–i.^48^ In the study of neurocytes, the cellular nanointerface between nerve cells and nanostructure arrays can promote the growth and number of axon collateral branches significantly. The physical interactions on the cellular nanointerface between the nanostructure arrays and the neurons can also induce cytoskeletal changing that give rise to the formation of lateral filopodia at the axon shaft, ultimately leading to the formation of axonal branches.^110^

The cellular nanointerfaces with different arrangements, geometries, and sizes of nanoarrays can not only provide guidance for the directional differentiation and directional growth of dendrite axons but also induce a specific morphology of neurons and can even improve the differentiation rate of nerve cells.^111^ For example, Aizenberg's group analyzed the effects of the ordered nanoarrays' radius, height, and interpillar spacing on stem cells systematically. The result indicated that under the induction of cellular nanointerfaces of the ordered nanoarrays with different shapes, the stem cells were induced to cells with either flattened, polarized, or stellate morphologies.^101^ In addition, the cells' morphology, behavior, and differentiation can also be directed by controlling the cells' adhesion.^103^ For example, Kelley's group exploited modified potential-responsive surfaces functionalized with tailored monolayers, which could tune the adhesion states and the differentiation of cells. More remarkably, this osteogenic differentiation was purely triggered by the dynamic tuning of adhesion molecules without the help of any cytokine or growth factor,^22^ as shown in Fig. 7j–l. Understanding the cellular nanointerface behavior is important for the precise control of cellular behavior, like the behavior of cellular morphology, the membrane curvature, cellular proliferation, apoptosis, and gene changes in cells.^10,97^

### 3.2 Cell safety study

#### 3.2.1 Cellular interface before penetration

Cellular proliferation, intracellular gene change, and other cellular behaviors at the nanointerface between cells and nanostructures before and after penetration are reviewed. In general, the cells cultured on vertical nanostructures often exhibit lower proliferation rates than those cultured on flat substrates.^8,10^ It has been discovered that the density of nanostructures will influence the following four cellular parameters, including the cells' viability, adhesion, morphology, and division. The presence of the convex structure of nanoarrays mainly influences the cells' division progress by increasing the number of cells in the S-phase and promoting cells to proceed through M- and cytokinesis phases in the cell cycle.^105^

#### 3.2.2 Cellular interface after penetration

Researchers have found that the nanostructure-mediated cell penetration exhibits excellent biosecurity, even with various materials and shapes of nanostructures. The vertical nanostructures were observed to not significantly affect the cellular activation or cellular proliferation.^112^ Based on the studies described above, researchers explored the ways for the cell membrane penetration assisted by external forces, including the mechanical force, electric field, magnetic field, light, heat, *etc.* Moreover, the chemical modification method can also create transient pores on the cell membrane.^49,82,113–118^ Large numbers of studies have shown that as long as the intensity of the external force is well controlled, the cellular perforation can be achieved with high cellular vitality. For example, Chen's group obtained diamond nanoneedle arrays by way of centrifugation-induced supergravity, which realized drug and biomaterial delivery into the cytosol and do not induce noticeable cytotoxicity.^8^ In addition, Elnathan's group prepared conical Si nanowire (NW) arrays for gene transfection. The proliferation capacity of cells on these conical Si NWs was similar to that of cells on flat Si, and it induced minimal necrosis and negligible apoptosis.^12^ In addition to the surface modification-induced cellular membrane perforation,^119^ laser,^37,59^ electricity,^120^ and many other stimuli under some certain conditions can also be used to achieve the intracellular penetration with high efficiency and good cellular activity. The cellular nanointerfaces with various topographies cause slight effects on cells' function besides intracellular delivery. These research studies on cellular interfaces will be helpful in guiding the designation of nanostructures with various biomaterials and provide inspiration on the development of many intracellular applications in sensing or drug delivery.^11,121,122^

### 3.3 Cellular immune activation and regulation

The cellular interaction between cells and the nanostructures is complex and dynamic. The nanoscale cellular interface enhances the interaction between the intracellular biomolecules and the bio cargoes outside the cell, allowing the bidirectional information interaction inside and outside of the cell. So, researchers can orient the cells' behavior and the cells' function by adjusting the surface structure and the properties of the cellular interface. For example, the cellular immune performance can be activated *via* cells' deformation induced by high aspect ratio nanoarrays, or the chemical modification of nanostructures, as well as the intracellular gene editing and modification.

#### 3.3.1 Cellular immune activation and regulation before penetration

**Fig. 8:**
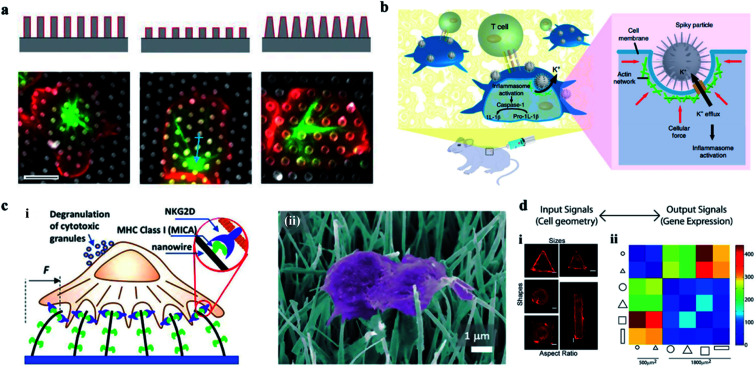
(a) The effect of a 3-D structure on T cell activation. Images illustrate the microtubule structure (green, β-tubulin) and cell morphology (red, CD45). Scale bar, 5 μm.^126^ (b) Schematic of spiky particles applied to activate immune cells and amplify immune responses *in vivo*.^74^ (c) (i) Schematic drawing of NK activation on MICA-functionalized nanowires. (ii) SEM image of NK cells on MICA-functionalized nanowires.^132^ (d) Cell geometry imposes modular changes in gene expression. (i) NIH 3T3 fibroblast cells cultured on fibronectin coated patterns of different sizes, shapes, or AR (AR is defined as cell adhering to micropatterns of similar area but different ratios of short axis to long axis.) (ii) Two-dimensional matrix showing the total number of differentially regulated genes among cells of different geometries.^134^ Reprinted with permission Copyright 2019, PNAS; Nature Nanotechnology 2018; Advance Materials 2019; 2013, PNAS.

The high aspect ratio nanostructures have been developed as a tool to induce cells' deformation. The cells' deformation could induce the cellular immune activation *via* the cytomembrane deformation and the rearrangement of the local cellular components such as the actin network and the membrane related proteins. The above response may further lead to the conformational changes of ion channels and the cascade of immune responses.^123–125^ The ability of immune responses can be regulated *in vivo* and *in vitro*. *In vitro*, depending on different cellular nanointerfaces, the cellular nanointerface increases the cell-specific endocytosis sites, which will activate the formation of T cells' immune synapses (Fig. 8a).^126^ It is known that endocytosis is a key and initial step for immune cells to perceive and clear pathogens. While the increased cell membrane curvature induced by nanoarrays could lead to enhanced endocytosis.^71^ Therefore, precise manipulation of the cytomembrane curvature *via* regulating the cellular interface may be a promising immunomodulation in cells.^32,127,128^ In addition, the curved cytomembrane could also create mechanical stress at the cellular interface. Under the action of mechanical forces induced by nanostructures, the cytoskeleton will deform and rearrange to activate a variety of corresponding cellular signaling pathways.^129,130^ Because the immune cells are sensitive to mechanical forces, the immune cells can activate the inflammatory microsomes and the other immune pathways by sensing the mechanical channel deformation and the cytoskeleton mechanical stress.^131^ The studies on the mechanical force in the immune system are an emerging research topic, and mainly focus on T cells and B cells to regulate the cellular immune function. For example, Xie's group prepared TiO_2_ spiky particles that could introduce mechanical stress on the cell membrane during phagocytosis, which leads to the inflammasome and cell pathway activation and significantly bolsters the T-cell and humoral immune responses, as shown in Fig. 8b.^74^ Schvartzman's team found that these forces not only come from different sources, such as actin dynamics, but also play important roles in different stages of lymphocytes' immune activity, as shown in Fig. 8c.^132^

Therefore, vertical nanostructures can be designed with some programmed topological features and surface functions. Cells at the customized cellular interface are likely to achieve some customized cellular immune transformation and activate some corresponding cellular immune function.^133^ In addition, the mechanical forces applied on the nucleus can also change the epigenetic regulatory networks, regulate the genes' expression, and ultimately activate the cellular immune function (Fig. 8d).^134^ For example, studies on the activation of immune response by acupuncture particles have confirmed the applicability of nanowire-mediated immune regulation *in vivo*.^135^ Thus, the cellular interface platforms characterized by nanostructures can be developed as safe tools for manipulating a wide range of immune cell activities.^136–140^

In addition to affecting the morphologies of immune cells,^140^ the morphologies of the cellular interface can also be designed to regulate the differentiation of immune cells.^141^ For example, Regev's group prepared a molecular circuit that can control the differentiation of mouse TH17 cells. Their research provided a basis for understanding how signals inside and outside the cell are processed into coherent cellular responses and determine the fate of cells.^52^ The chemical modification of nanostructures at the cellular nanointerface has also been used to regulate cellular immune function. For example, Schvartzman *et al.* have reported nanowires covered with a receptor and/or ligand to activate the specific target cells, which can further enhance the ability of nanowire-mediated immune regulation.^142^

#### 3.3.2 Cellular immune activation and regulation after penetration

**Fig. 9:**
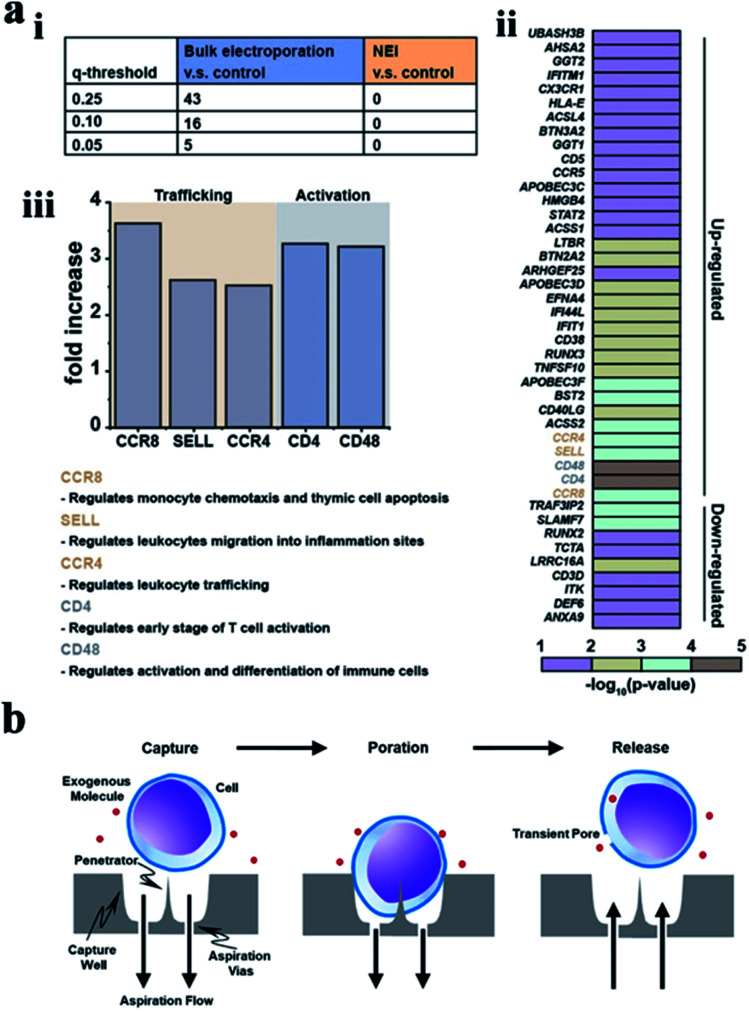
(a) Bulk electroporation led to unintended and nonspecific expression changes in immune-associated genes. (i) Table showing the number of immune associated genes with altered expressions due to bulk electroporation with different FDR *q*-values. (ii) Heatmap of immune-associated genes affected by bulk electroporation. 43 genes were affected with FDR *q*-value < 0.25. (iii) Five genes (FDR *q*-value < 0.01) related to immune cell trafficking (CCR8, SELL, and CCR4) and activation (CD4 and CD48) were affected by bulk electroporation.^145^ (b) Schematic of a single cell capture site using negative aspiration flow.^147^ Reprinted with permission Copyright 2019, Advanced Therapeutics; 2019, Nano Lett.

The intracellular gene transfection is a key step in intracellular gene editing and cell therapy. The activation of cellular immunity by gene transfection requires puncturing the cytomembrane at the cellular interface.^143,144^ Melosh's lab developed a nanometer electric injection (NEI) platform combined with the electrophoretic technology for efficient intracellular DNA delivery. This platform uses local electric fields to briefly open holes on cell membranes that can transfect primary immune cells and stem cells, generating minimal perturbation of gene expression and negligible Ca^2+^-mediated cellular stress. Moreover, only the immune-related genes involved in the activation and transport of immune cells were expressed significantly, as shown in Fig. 9a.^145^ These results highlight the advantages of nanoscale cellular interface delivery in cell-based research and the immunotherapy applications from the perspective of cell health. In addition, the chemical modification can also achieve specific recognition and capture of cells to be transfected or transformed.^146^ With the help of the microfluidic equipment, the negative pressure air flow can be used to capture a single cell for genes' transfection in the immune modification project (Fig. 9b).^147^

The activation and transport of immune cells are an effective means for tumor immunotherapy. The surface functionalization and the external forces applied on nanostructures could enhance the interfacial interaction between the cells and the nanostructures. These studies on the cellular interface provide an important reference for the realization of cellular immune activation, the regulation of immune cells with a targeted function, the precise capture of tumor cells, and further immune activation, as well as oriented genes' transfection in immune cells and the targeted therapy for the treatment of solid tumors.^148–150^

Studies on the immune cells' behavior with or without external forces' stimulation have an extremely important reference value on the cellular immunotherapy, further potential tumor therapy, and other disease treatments.^151,152^ The immune cells' behavior mainly includes the cellular immunity effect, the duration of the immune function, and whether the immune function and genes’ transfection affect the other normal cellular functions.^151,152^ Research on the shape, size, geometry, hardness, and chemical modification of different nanostructures, as well as the comprehensive and systematic research on the immune function of immune cells at the customized cellular interface with or without the stimulation of external factors such as light, electricity and other external forces, can provide guidance value for the future immunotherapy of tumor and other diseases.^153,154^

## 4. Applications

Studies on the cellular nanointerface have great significance in obtaining the environmental information inside and outside the cell. At present, many research works have shown that the application of cellular nanointerfaces with vertical nanostructures has achieved promising achievements in different cellular aspects, such as drug delivery, detecting intracellular and extracellular electrical signals, recording biochemical information, capturing cancer cells, and manipulating cell behavior.

### 4.1 Drug delivery

It is possible for the vertical nanostructures to penetrate the cell membrane and get access to the intracellular environment, especially when physical stimulations are coupled to the structures. Although the question of whether the nanostructures can penetrate cell membranes has been controversial, a series of research works on successful penetration with drug delivery into cells have been demonstrated. Traditional models usually require some transfection reagents to initiate cell endocytosis, with issues of drug degradation, low efficiency, and high cytotoxicity. Whereas the nanoneedle-mediated delivery can bypass degradation pathways and introduce a wide range of materials into a variety of cell types, including those that are difficult to transfect.

**Fig. 10:**
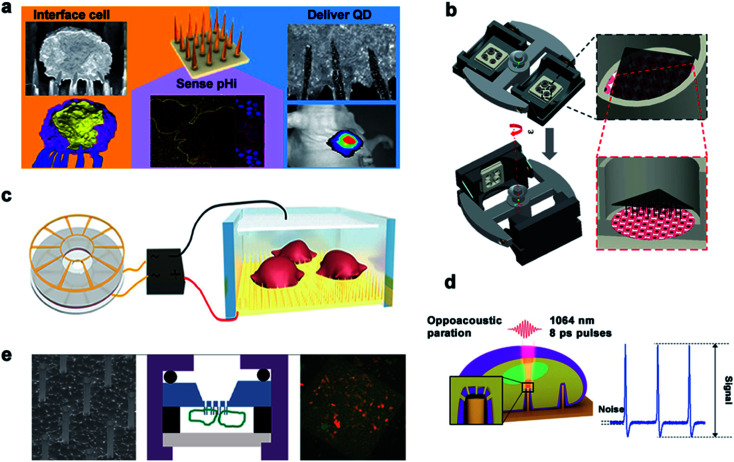
Main delivery methods. (a) Schematic diagram of spontaneous drug delivery. (b) Schematic for gene transfection by centrifugal force. (c) TENG-driven electroporation systems. (d) Electrophysiological diagram of photoacoustic perforation on the MEA configuration of nanostructures. In the laser focusing volume, the photoacoustic hole is locally generated in the membrane. (e) Schematic diagram of drug delivery by chemical action. Reproduced with permission from ref. 3, 156, 157, 159 and 160 Copyright (2015) American Chemical Society Publishing Group, (2014) Nature Publishing Group, (2019) Advanced Materials Publishing Group, (2019) Advanced Biosystems Publishing Group and (2012) American Chemical Society Publishing Group, respectively.

Up to now, there are five main types of transmembrane methods for drug release through nanoneedles, as is shown in Fig. 10. First, nanoneedles can deliver drugs into the cell in a spontaneous manner. As reported by Nir Yosef *et al.*, they used a silicon nanowire-based delivery technique to assist in the construction and validation of a network model for TH17 differentiation by spontaneously enabling it to deliver siRNA efficiently without activating T cells.^155^ Besides, Ciro Chiappini *et al.* reported a mesoporous silicon nanoneedle array that can nanoinject quantum dots into the cytosol to form a tight interface with cells and quickly pass through a local biological barrier.^156^ The second is about the mechanical force of the nanoneedle. Utilizing a new platform for diamond nanoneedle arrays, membranes deformed by a few nanometers' force of nanoneedle arrays can transfer large molecules and materials to different kinds of cells.^157^ With the help of centrifugal force, Yaping Chen *et al.* developed a gene transfection platform for silicon nanoarrays to transfect adherent cells and non-adherent cells *in vitro*. Third, nanoneedles deliver the drug through electricity-mediated cell perforation. In previous studies, Xie *et al.* demonstrated a simple nanoscale electroporation platform that successfully delivers impermeable dyes and plasmids to cells through alumina nanotubes and low-voltage pulses as switches while maintaining a high cell survival rate.^158^ Combined with the silicon nanoneedle array electrode, intracellular drug delivery could be performed with high efficiency and minimal cellular damage by enhancing local electric fields at the cell interface.^159^ The fourth method is achieved by nanostructure-coupled optoporation. Gold nanotubes are attached tightly to the cells on one side and connected to the microchannels on the other side. The nano-shock waves opened the transient nanopores in the cell membrane at the end of the nanotube and delivered the desired molecules to the selected cell.^104^ Michele Dipalo *et al.* demonstrated the cellular dynamic response of plasma photoacoustic perforation of three-dimensional nanostructures on MEAs. The CAAX membrane protein was transferred to study how the plasma membrane rearranges the conformation according to the pore formation.^3^ The fifth method is by chemical sensing. A hollow nanoneedle array system is used in which nanoneedles are fed by a common reservoir. By combining the mechanical action of the nanometer on the cell membrane with the local concentration of the reagent saponins, different cargos can be transported to the same cell at the same time.^160^

### 4.2 Electrical signal recording

Recording the electrical signals of excitable cells can be used to study and judge the physiological state of cells. At present, there are two types of cell electrical signal recording: extracellular recording and intracellular recording. Traditional extracellular detection usually uses a two-dimensional structured device, such as a field effect transistor (FET)^161^ and a microelectrode array. For the detection of intracellular electrical signals, a patch clamp – the gold standard technique – can accurately record the amplitude and the shape of electrical signals under high signal-to-noise ratio conditions. However, due to its invasiveness and complexity, the patch clamp has a short recording time and cannot record signals from multiple cells at the same time. With the development of micro-/nanotechnology, the quality of electrical signals recorded inside and outside the cell can be greatly improved by establishing a three-dimensional cell–nano interface.

**Fig. 11:**
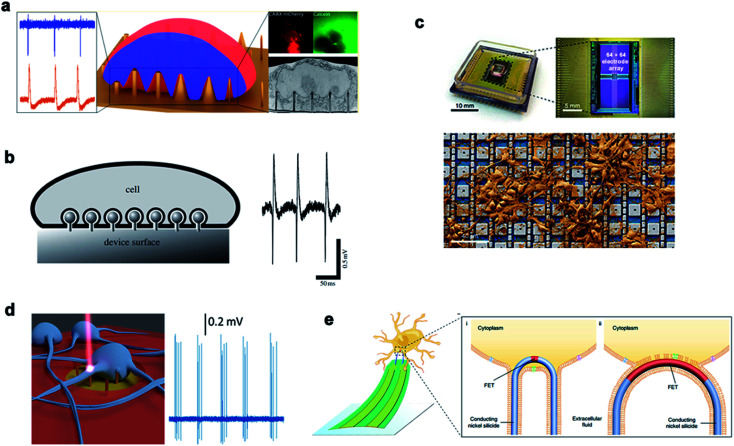
Nanoneedles record electrical signals. (a) Measurement of external electrical signals of different nanostructures. (b) The electrodes and cells record extracellular signals by phagocytosis. (c) A physical image of a CMOS activated PtB electrode array encapsulated by electrical stimulation for recording. (d) Schematic diagram of the 3D nanometer electrode recording extracellular signals after laser excitation. (e) Schematic diagram of the nanoneedle modified chemically to bind to the cell membrane and record signals. Reproduced with permission from ref. 162, 163, 165, 166 and 167 Copyright (2018) Nano Letters Publishing Group, (2009) The Royal Society Interface Publishing Group, (2019) Nature Publishing Group, (2017) Nano Letters Publishing Group and (2019) Nature Publishing Group, respectively.

Various nanostructures are used to measure the potential of cardiomyocytes and nerve cells. The dynamic interface between the cell membrane and the nanostructure determines the biological process and the recorded signal quality. Michele Dipalo *et al.*^162^ reported in their study that nanostructures could tightly couple to the cell membrane, leading to the formation of a stable nano–bio interface. While maintaining the integrity of the cell membrane, the cell membrane could closely adhere to nanostructures of various shapes and enable the recording of extracellular electrical signals. Inspired by the phagocytosis of living cells, Aviad Hai *et al.*^163^ developed a three-dimensional electrode structure phagocytosis, which formed a good seal with the cells and increased the electrical coupling of neurons. The three-dimensional electrode structure had a large surface area, which reduced the electrode impedance and significantly increased the field potential signal (Fig. 11).

Chong Xie^45^ and Hongkun Park^164^ fabricated Pt electrodes and Pt/SiO_2_ electrodes, respectively, to achieve membrane penetration through electroporation and recording of the intracellular action potential. For a large number of neural networks, it is necessary to test neural electrical signals with a high flux. A nano-electrode array could acquire the potential of thousands of connected neurons at the same time and measure the effects of drugs through the ion channel current. This high-throughput intracellular recording technique could be used for electrophysiological screening of neural networks.^165^ In addition to electroporation to achieve transmembrane, 3D plasma electrodes can also monitor the intracellular potential through coupling with optoporation. Michele Dipalo *et al.* reported a combination of vertical nano-electrodes and plasma optics to record the intra- and extra-cellular electrical stimulation activities of primary mammalian neurons and HL-1 cells. The plasma photochemistry did not interfere with spontaneous electrical activity and had a good signal-to-noise ratio.^166^ In recent years, Lieber had also constructed various kinds of nanoprobes for the application of 3D nanoprobes in neural electrical signal impulses and realized high-throughput synchronous monitoring from extracellular to intracellular and from single cells *in vitro* to large-scale neural network tissues *in vivo*. Coupling and fusion of cell–nano interfaces are generally achieved by coating nanoprobes with a phosphate bilayer. Zhao *et al.* studied how the size and the geometry of the nanoprobe affected intracellular recording by fabricating scalable three-dimensional U-shaped nanowire field effect transistor arrays. The nanowire arrays modified with phospholipids recorded better intracellular action potentials and subthreshold characteristics at a full amplitude.^167^

### 4.3 Biochemical signal recording

Real-time regulation and monitoring of intracellular activities are of great significance for disease investigation and deciphering the pathogenesis of diseases related to cell behavior. Traditional methods, such as cell content sequencing techniques and single-cell mRNA and protein detection, require cell lysis to extract cytoplasm. However, these methods allow detection only at a single point in time, without the feasibility of real-time intracellular monitoring and cannot improve the information of previous or future states. In recent years, nanoneedle arrays have successfully delivered exogenous substances in the form of nucleic acids, proteins, metabolites, and cell-impermeable nanoparticles. The intimate interface with the cell membrane enables the nanoneedle to sense proteins, metabolites, and lipids in the intracellular environment, thus monitoring cell dynamics in real time.

**Fig. 12:**
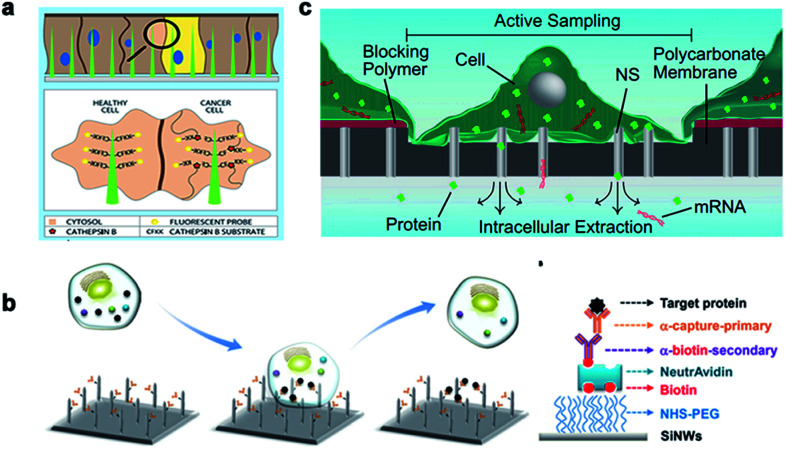
The nanoneedles record biochemical signals. (a) The nanoneedle is in contact with the cytoplasm, where the active CTSB lyses its CFKK peptide substrate, thereby releasing the attached TAMRA fluorescent probe into the cytoplasm of the cell. (b) Schematic diagram of the silicon nanoneedle surface modified by the target protein. (c) Schematic diagram of intracellular substances diffusing through the nanostraw into the buffer below the membrane to extract mRNA and detect it. Reproduced with permission from ref. 168, 169 and 170 Copyright (2015) Advanced Materials Publishing Group, (2016) Royal Society of Chemistry and (2017) Proceedings of the National Academy of Sciences Publishing Group, respectively.

Nanoneedles can detect enzyme activity, such as that of cysteine protease CTSB, which is a widely used biomarker for solid tumors. Nanoneedle sensors can identify CTSB positive (+ve) cancer cells and CTSB negative (−ve) cancer cells in mixed cultures. As a minimally invasive detection tool, nanoneedles allow biopsy sites to be more precise, leading to earlier detection and treatment of lesions. Nanoneedles can also detect intracellular proteins through surface-modified antibodies. In recent research, it was reported that silicon nanoneedle arrays directly entered the intracellular cavity of living cells, which was simple and effective for detecting specific endogenous target protein complexes in suspended cells and did not require isolation by destroying cells. Endogenous proteins could be observed without the need for additional tags or modifications.^168,169^ Furthermore, electroporation can assist the nanoneedle to extract mRNA and detection. Cao *et al.* reported a method for the quantitative measurement of mRNA for multiple cell types. Assisted by a voltage system, the nanostraw extraction system was non-destructive and provided intracellular mRNA sequences and proteins. The extracted molecules were separated from the cell culture and collected simply using a pipette or microfluidic devices, where the cell contents were analyzed using conventional methods^170^ (Fig. 12).

### 4.4 Cancer cell capture

**Fig. 13:**
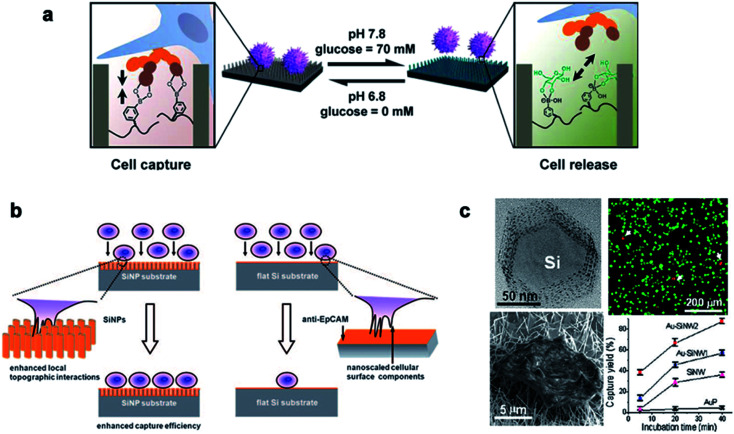
(a) Cell capture and release by pH and glucose induction. (b) Left: improving the capture efficiency: the staggering of nano-scale cell surface components and SiNPs enhances the local terrain interaction; right: impacting the capture efficiency: lack of the local terrain interaction between cells and a flat Si substrate. (c) The fluorescence images of the cells on the Au–SiNW_2_ substrate, and the cell capture rate on different substrates increased with the incubation time. Each error bar represents the standard error of the mean. Reproduced with permission from ref. 171, 173 and 174 Copyright (2013) Journal of The American Chemical Publishing Group, (2009) Wiley InterScience Publishing Group and (2012) Nano Letters Publishing Group Publishing Group, respectively.

Traditional detection methods do not easily quickly and effectively detect rare cells such as CTCs, which limit the early diagnosis and development of malignant tumors and delay the best time for treatment to a large extent. In recent years, nanowire arrays have been proposed to capture and analyze cancer cells.^171,172^ Through the role of the cell–nano interface, the capture ability of the cell is improved. In his research, Wang *et al.*^173^ demonstrated that the capture efficiency of the cell–nano interface can be improved by 3–10 times. In addition, Park *et al.*^174^ and Liu *et al.*^172^ reported that the ability to capture breast cancer cells can be effectively improved by improving nano-surface coatings and nano-surface materials, respectively (Fig. 13). All of these reports show the ability of nanoparticles to capture cancer cells.

### 4.5 Cell behavior manipulation

Many studies have shown that the cell–nano interface influences the cell behavior, including the cell migration, differentiation, mechanical force transduction, and so on. Therefore, the cell–nano interface can be employed to manipulate various cell behaviors and continue to advance the development of tissue engineering and regenerative medicine.

**Fig. 14:**
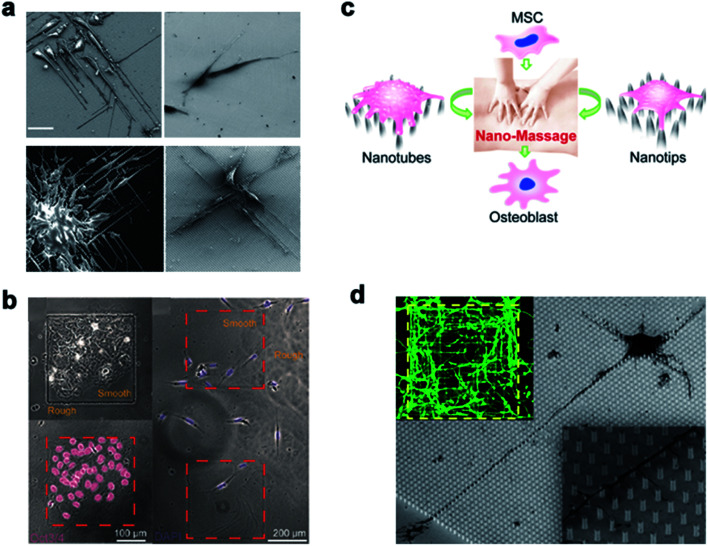
(a) Induced polarization of cells by NP arrays of different sizes. The cells were cultured for 1 day and observed with a scanning electron microscope. Scale bar, 50 μm. (b) On the left, Oct3/4 + hESCs are selectively attached and aggregated on the smooth areas of the patterned nanoglass substrate. The picture on the right shows the preference of Oct3/4-hESCs randomly distributed on the patterned nano-roughened glass surface. After the cells were cultured for 48 hours, the nuclei (color) Oct3/4 (red) were treated with DAPI. (c) Schematic representation of nanotube/nanotip transitions on a Ppy array. (d) Cortical cultured neurons after 2 DIV. The inset is a close shot around the axon. Reproduced with permission from ref. 27, 101, 175 and 176 Copyright (2012) American Chemical Society Publishing Group, (2012) American Chemical Society Publishing Group, (2017) American Chemical Society Publishing Group, and (2017) Nano Letters Publishing Group, respectively.

Michael A. Bucaro *et al.*^101^ adjusted the geometry of the ordered array of nanoparticles (NPs) to induce the special morphology of adherent cells. These special geometries supported the ability of stem cells to form various morphogenetic trends, thereby providing a dynamic environment for cell growth. Weiqiang Chen *et al.*^175^ and Yan Wei *et al.*^27^ manipulated hESCs and osteoblast differentiation through the cell–nano interface, respectively, and provided the basis for the co-culture system of tissue engineering and intelligent biological interface design. In addition, Vini Gautam *et al.*^176^ demonstrated for the first time that the isotropic arrangement of indium phosphide (InP) nanowires could serve as a physical clue to guide the growth of neurites and help form a network with neighboring neurons. Under the guidance of the InP nanowire scaffold, the neuronal processes of multiple neurons showed synchronized calcium activity. Using these nanowires to design the growth of neurons could eventually develop nerve repair techniques in regenerative medicine. Waldemar *et al.*^177^ used epitaxially grown monodisperse nanowire arrays to measure the spatial resolution of cells. Nerve cells were cultured on the array, and the cell force was calculated from the displacement of the nanowire tip. The research on the growth cone dynamics and axon regeneration has great prospects (Fig. 14).

## 5. Summary and perspective

To summarize, with its versatility and biocompatibility, vertical nanostructures allow manipulation of cells with minimal invasiveness. Because of their unprecedented spatial and mechanical resolution, vertical nanostructures presented great capability to coordinate cell behavior. The minimal invasiveness nature of vertical nanostructures offers promising opportunities for cargo transport into cells, where vertical nanostructures have been proved to effectively deliver a series of biological molecules into primary cells that are difficult to be transfected, overcoming the low efficiency, high potential, and cellular safety problems compared with the traditional virus and non-virus methods. Moreover, vertical nanostructures may even serve as innovative tools for the long-term monitoring of intracellular biochemical information or recording of electrical signals of excitable cells (*e.g.*, neurons, cardiomyocytes). The versatile features of vertical nanostructures make them a promising platform for cell manipulation and cell monitoring that would benefit the fundamental biomedical research.

There are also several key challenges to be addressed in future. First of all, with the rapid expansion of the multimodal cellular nanotechnology, developing new, low-cost, and easy to realize nanomanufacturing routes will be the key to seamless integration of nanostructure-based devices and their subsequent application in the biomedical field. Second, the cell behavior at the nano-interface between cells and vertical nanostructures is highly complex due to the difference in cell types and properties of nanostructures, such as the surface functionalization, the chemical modification and composition, the electronic properties, and the physical morphology of nanostructures. Therefore, the understanding of how to utilize these structural variations to regulate cells would enable vertical nanostructures as a robust platform to mediate cell behaviors. Third, combining the powerful characterization techniques (such as the low temperature electron microscopy, scanning ion conductivity microscopy, and electron tomography) and their corresponding theoretical models, the detailed correlation of the collaborative parameters at the cellular nanointerfaces and a further understanding of the mechanism of cell membrane penetration by nanostructures remain to be explored. Further advances in biological imaging will allow *in vivo* real-time monitoring of molecular and cellular signaling pathways regulated by vertical nanostructure–cell interactions.

## Conflicts of interest

There are no conflicts to declare.

## Supplementary Material
